# When do you not listen to your doctor

**DOI:** 10.11604/pamj.2019.32.125.16062

**Published:** 2019-03-15

**Authors:** Catia Aguiar Cabral, António Grilo Novais

**Affiliations:** 1 Internal Medicine Service, Centro Hospitalar Tondela-Viseu, Viseu, Portugal

**Keywords:** Gout, diet, medication

## Image in medicine

Gout is a disease caused by an inflammatory response to crystals of sodium monourate, which occurs in people with elevated uric acid levels. There are acute and chronic forms. Acute forms arise as sudden, self-limiting bouts of arthritis (swelling, flushing, pain and heat of a joint), while chronic forms result in the deposition of crystal aggregates in and around the joints, with progressive joint destruction. In addition to joint manifestations, uric gout has renal manifestations (kidney stones and renal insufficiency) and metabolic manifestations (hypertension, elevated triglycerides). Patients are often obese, have high alcohol consumption and insulin resistance. It is a very painful and disabling illness. Gout is more common in men and usually begins in the 40's and 60's. Analytically the patient had uric acid values of 10.2 mg/dL (2.0-7.0 mg/dL) despite being daily medicated with alopurinol 300mg for more than 5 years. It has an abnormality of fasting glycemia and triglycerides of 305 mg/dl. However it maintains consumptions of alcohol and a diet rich in meat, seafood and fats. He did not diet to lose weight and sometimes he does not take the medication (alopurinol). He already shows involvement and destruction of the joints and deformation on the feet and the hand that currently prevent him from fulfilling the necessary safety norms in his profession.

**Figure 1:**
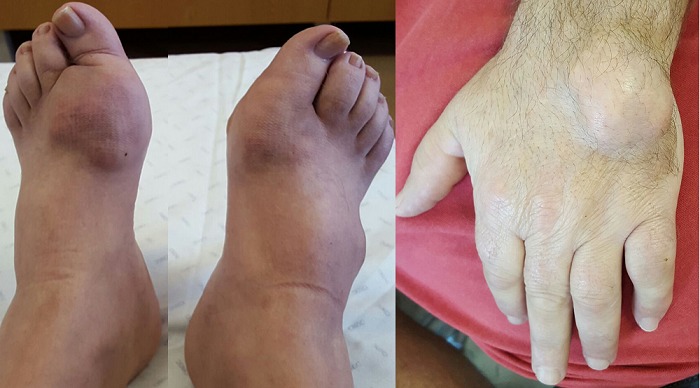
(A,B) this patient was advised several times by the doctor that he had to change his diet but he did not fulfill the recommendations

